# Low‐dose caffeine administration increases fatty acid utilization and mitochondrial turnover in C2C12 skeletal myotubes

**DOI:** 10.14814/phy2.14340

**Published:** 2020-01-21

**Authors:** David S. Enyart, Chelsea L. Crocker, Jennifer R. Stansell, Madeleine Cutrone, Meghann M. Dintino, Stephen T. Kinsey, Stephan L. Brown, Bradley L. Baumgarner

**Affiliations:** ^1^ Division of Natural Science and Engineering University of South Carolina Upstate Spartanburg SC; ^2^ Department of Biology and Marine Biology University of North Carolina Wilmington Wilmington NC; ^3^ Department of Cell Biology and Physiology Edward Via College of Osteopathic Medicine Spartanburg SC

**Keywords:** caffeine, lipid utilization, mitochondrial turnover, mitophagy, skeletal muscle

## Abstract

Caffeine has been shown to directly increase fatty acid oxidation, in part, by promoting mitochondrial biogenesis. Mitochondrial biogenesis is often coupled with mitophagy, the autophagy‐lysosomal degradation of mitochondria. Increased mitochondrial biogenesis and mitophagy promote mitochondrial turnover, which can enhance aerobic metabolism. In addition, recent studies have revealed that cellular lipid droplets can be directly utilized in an autophagy‐dependent manner, a process known as lipophagy. Although caffeine has been shown to promote autophagy and mitochondrial biogenesis in skeletal muscles, it remains unclear whether caffeine can increase lipophagy and mitochondrial turnover in skeletal muscle as well. The purpose of this study was to determine the possible contribution of lipophagy to caffeine‐dependent lipid utilization. Furthermore, we sought to determine whether caffeine could increase mitochondrial turnover, which may also contribute to elevated fatty acid oxidation. Treating fully differentiated C2C12 skeletal myotubes with 0.5 mM oleic acid (OA) for 24 hr promoted an approximate 2.5‐fold increase in cellular lipid storage. Treating skeletal myotubes with 0.5 mM OA plus 0.5 mM caffeine for an additional 24 hr effectively returned cellular lipid stores to control levels, and this was associated with an increase in markers of autophagosomes and autophagic flux, as well as elevated autophagosome density in TEM images. The addition of autophagy inhibitors 3‐methyladenine (10 mM) or bafilomycin A1 (10 μM) reduced caffeine‐dependent lipid utilization by approximately 30%. However, fluorescence and transmission electron microscopy analysis revealed no direct evidence of lipophagy in skeletal myotubes, and there was also no lipophagy‐dependent increase in fatty acid oxidation. Finally, caffeine treatment promoted an 80% increase in mitochondrial turnover, which coincided with a 35% increase in mitochondrial fragmentation. Our results suggest that caffeine administration causes an autophagy‐dependent decrease in lipid content by increasing mitochondrial turnover in mammalian skeletal myotubes.

## Graphical abstract

Low‐dose caffeine promoted autophagy in skeletal myotubes. Inhibiting autophagy reduced caffeine‐dependent lipid droplet breakdown by 30%. Caffeine did not directly increase lipophagy in skeletal myotubes.

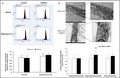

## 1 INTRODUCTION

The prevalence of obesity has risen dramatically in the United States over the past three decades (Hurt, Kulisek, Buchanan, & McClave, 2010). Obesity can result in increased cellular lipid accumulation (lipid droplets) in numerous mammalian cell types, including skeletal muscle (Bosma, Kersten, Hesselink, & Schrauwen, 2012; Sinha et al., 2014). Macroautophagy (hereafter referred to simply as autophagy) is the pathway by which eukaryotic cells degrade long‐lived proteins, organelles, and bits of cytoplasm in a lysosomal‐dependent manner (Reggiori & Klionsky, 2002). Autophagy is a highly conserved and tightly regulated process that involves the formation of double membrane vesicles (autophagosomes) around molecules that have been targeted for degradation (Wang & Klionsky, 2003). Recent studies have revealed that cellular lipid droplets can be specifically utilized in an autophagy‐dependent manner in certain mammalian cell types, a process referred to as lipophagy (Liu & Czaja, 2013). However, the role of lipophagy in regulating skeletal muscle lipid metabolism has yet to be fully investigated.

Mitochondria are targeted for lysosomal‐dependent degradation through the process of mitophagy, which serves an essential role in mitochondrial turnover by removing damaged or dysfunctional mitochondria (Cui et al., 2018; Wu et al., 2015). Mitochondrial turnover also requires increased mitochondrial biogenesis (Sin et al., 2016). Endurance exercise has been shown to increase mitochondrial turnover in skeletal muscle by increasing mitochondrial biogenesis and the rate of mitochondrial degradation (Vainshtein, Tryon, Pauly, & Hood, 2015). In addition, increased mitochondrial metabolism has been directly linked to increased mitochondrial turnover in human skeletal muscle cells (Twig et al., 2008). It remains unclear whether dietary stimulants, such as caffeine, can promote mitochondrial turnover in skeletal muscle cells.

Caffeine has been shown to be a potent activator of cellular lipid metabolism. A previous investigation revealed that caffeine administration increased whole‐body lipid metabolism and reduced fat mass (Guarino, Ribeiro, Sacramento, & Conde, 2013; Ohara, Muroyama, Yamamoto, & Murosaki, 2016; Panchal, Wong, Kauter, Ward, & Brown, 2012; Wu et al., 2017). Although caffeine was recently demonstrated to increase cellular lipid utilization in a lipophagy‐dependent manner in murine hepatocytes (Sinha et al., 2014), it remains unclear whether caffeine can promote lipophagy in skeletal muscle cells. Our laboratory previously demonstrated that higher caffeine doses (2.5–10 mM) significantly increased autophagy in C2C12 murine skeletal myotubes (Hughes et al., 2017; Mathew, Ferris, Downs, Kinsey, & Baumgarner, 2014). However, it remains unclear whether lower, more physiologically relevant doses of caffeine can significantly increase autophagy, and possibly lipophagy, in skeletal muscle cells. Furthermore, caffeine has been shown to increase mitochondrial biogenesis in skeletal myotubes treated with higher caffeine doses (5–10 mM) (Ding, Contrevas, Abramov, Qi, & Duchen, 2012). The effect of physiologically relevant doses of caffeine on mitochondrial turnover has yet to be fully investigated. The goal of this investigation was to determine whether a physiologically relevant dose of caffeine could significantly increase lipid utilization in lipophagy‐dependent manner skeletal myotubes. In addition, we sought to determine whether a physiologically relevant dose of caffeine could increase mitochondrial turnover in skeletal myotubes.

## 2 MATERIALS AND METHODS

### 2.1 Materials

Fetal bovine serum (FBS), horse serum (HS), penicillin/streptomycin (pen/strep), and Dulbecco's modified Eagle's medium (DMEM) were purchased from Life Technologies. Primary antibodies for microtubule‐associated protein 1 light chain 3b (LC3b) was purchased from Novus Biological. The primary antibody for carnitine palmitoyltransferase‐1/2 (CPT‐1/2) and the Total OXPHOS Rodent WB Antibody Cocktail (OXPHOS) were purchased from Abcam. The primary antibody for Perilipin‐1 was purchased from Cell Signaling Technology. All secondary antibodies were purchased from Vector Laboratories. All pharmacological agents were purchased from EMD Millipore.

### 2.2 Cell culture

C2C12 myoblasts were purchased from ATCC. Myoblasts were grown to confluence in normal growth media (DMEM plus 10% FBS, 100 U/ml penicillin, and 100 μg/ml streptomycin) at 37°C in a water‐saturated atmosphere of 5% CO_2_. To promote differentiation of mononucleated myoblasts into multinucleated myotubes, the media were switched to differentiation media (DMEM plus 2% HS, 100 U/mL penicillin, and 100 μg/mL streptomycin). Fully differentiated myotubes were normally present upon 5–6 d incubation in differentiation media.

### 2.3 Western blot analysis

Cell lysates were collected in lysis buffer (1 × TBS, 1% Nonidet P‐40, 0.5% sodium deoxycholate, 0.1% SDS, 0.004% sodium azide, 2 mM PMSF, 1 mM sodium orthovanadate, and 10 μl protease inhibitor cocktail), immediately snap frozen in liquid nitrogen, and stored at −20°C. All samples were vortexed and centrifuged at 15,000*g* for 15 min at 4°C and the supernatants were collected. The total protein concentration of each sample was determined using a Bradford assay. An equal volume of supernatant was combined with 2× Laemmli buffer and boiled for 10 min. Twenty micrograms of each denatured sample was submitted to SDS‐PAGE using either 7.5% or 12% polyacrylamide gels and subsequently transferred on to a PVDF membrane (EMD Millipore). All membranes were blocked for 1 hr in 5% bovine serum albumin (BSA) dissolved in Tris‐buffered saline plus 0.1% Tween 20 (TBST), incubated with primary antibody (1/1,000) over night at 4°C and subsequently labeled with an appropriate HRP‐labeled secondary antibody (1/10,000) for 1 hr at room temperature. Once satisfactory images were obtained, each membrane was stained with Coomassie brilliant blue (R‐250) for 5 min, washed in TBST, and imaged for total protein assessment. Unless otherwise stated, all blots were normalized to total protein. Blots were developed using standard ECL detection and images were acquired on a FluorChem E System imager (ProteinSimple). Digital images were analyzed using ImageJ 1.47v (National Institutes of Health).

### 2.4 Seahorse metabolic analyzer analysis

Cells were cultured and subjected to treatments as described above in a Seahorse XFp eight‐well microplate cartridge. Two of the wells were calibration controls that contained only media, and the remaining wells contained cells. The cellular oxygen consumption rate (OCR) was measured in a Seahorse XFp metabolic flux analyzer (Seahorse Bioscience). To evaluate mitochondrial function, OCR was measured in the presence of (a) only DM to measure basal rates, (b) 1 μM oligomycin to inhibit the ATP synthase and measure OCR from the mitochondrial proton leak and non‐mitochondrial OCR, (c) 2 μM FCCP to uncouple mitochondria and measure maximal OCR, and (d) 0.5 μM rotenone/antimycin A to inhibit the electron transport system and measure only nonmitochondrial OCR. To evaluate the relative contribution of fatty acids, glucose, and glutamine‐to‐oxidative metabolism, OCR was measured during application of inhibitors for each fuel. Fatty acids, glucose, and glutamine were inhibited by 4 μM etomoxir, 2 μM UK5099, and 3 μM BPTES, respectively. OCR was measured initially under baseline conditions, then following injection of the inhibitor for the target fuel, and finally after the addition of the other two fuel inhibitors. The relative fuel use was calculated as:

$$\begin{array}{cc} \%\text{fuel use} & =\left[\left(\;\text{OCR}-\text{target inhibitor OCR}\right)/\left(\text{baseline OCR}-\text{all inhibitors OCR}\right)\right]\\ & \;\times 100. \end{array}$$

### 2.5 Transmission electron microscopy

Cells were grown in six‐well plates, three control wells were treated with oleic acid for 24 h and three treatment wells were treated with oleic acid for 24 h plus 0.5 mM caffeine for 6 h. Cells were harvested and fixed in a modified Karnovsky's fixative (2.5% glutaraldehyde and 2.0% paraformaldehyde in 0.1 M Sorenson's phosphate buffer, pH 7.4) overnight at room temperature. Cells were rinsed in Sorenson's phosphate buffer, followed by secondary fixation in 1.0% osmium tetroxide with 0.8% potassium ferricyanide in 0.2 M cacodylate buffer, pH 7.4. Samples were rinsed and dehydrated with an ethanol series, and embedded in Spurr's epoxy resin (Electron Microscopy Sciences). Embedded samples were trimmed, and 90 nm sections were cut on a Leica UC7 ultramicrotome (Leica Microsystems, Inc.). Sections were collected on Formvar‐coated (0.25 g Formvar in 100 ml ethylene dichloride), 200 mesh copper grids. Sections were stained with 2% uranyl acetate in 50% ethyl alcohol for 15 min, rinsed with DI, and stained with Reynolds lead citrate for 15 min. Sections were imaged on a Tecnai 12 Spirit TEM (FEI). A total of 30 images were collected for each treatment group and images were analyzed in Fiji (Schindelin et al., 2012). Point counting stereological analysis was used to determine volume density of autophagosomes and mitochondrial.

### 2.6 Fluorescence microscopy

Myoblasts were seeded on 35 mm dishes that contained a 1.5‐mm‐thick collagen coated coverslip (MatTek Corporation) in normal growth media. Once the cells were 75% confluent, they were switched into differentiation media for 5–6 d to promote the formation of multinucleated myotubes. Triplicate groups of cells were exposed to vehicle (DMSO, control) or a combination of 0.5 mM oleic acid with 0.5 mM caffeine. All possible experimental scenarios were tested using these treatment groups. All samples were washed in TBS and fixed with 4% paraformaldehyde for 20 min. Nuclei were labeled with1 μl/ml of Hoechst 33342 nuclear stain for 10 min. Cells were permeated with 0.1% Triton X‐100 in TBS for 30 min and subsequently blocked with 1% BSA in TBS for 1 hr. All samples were incubated for 1 hr with 0.5% BSA in TBS containing a 1:250 dilution of primary antibody for Perilipin. Samples were analyzed using a Nikon Eclipse TS100‐F microscope and NIS Elements Br software (Nikon Instruments). For Perilipin‐1 analysis, images were captured in the TRITC channel using a 40× fluorite objective. Ten images were randomly captured on each coverslip (replicate sample), which were subsequently used to calculate the mean FITC intensity for each given sample and thus for each experimental treatment (*n* = 3). For colocalization analysis, images were captured in the FITC (LC3b or Perilipin‐1) and TRITC (Perilipin‐1 or LysoTracker Red) channels using a 40× fluorite objective. Ten images were randomly captured on each coverslip (replicate sample). Colocalization analysis was conducted using Coloc2 plugin for Fiji (ImageJ) software (National Institutes of Health). A Pearson's correlation coefficient was calculated for each image and all 10 correlation coefficients were used to calculate the mean correlation coefficient for each sample replicate (*n* = 3).

### 2.7 Oil red O analysis

Myoblasts were seeded on 24‐well collagen‐coated plates and grown to confluence. Once the cells were 75% confluent, they were switched into differentiation media for 5–6 d to promote the formation of multinucleated myotubes. Fully differentiated myotubes were exposed to vehicle (DMSO, control) or a combination of 0.5 mM oleic acid with 0.5 mM caffeine (*n* = 6). All possible experimental scenarios were tested using these treatment groups. All samples were washed in TBS and fixed with 4% paraformaldehyde for 20 min. Upon fixation, all cells were washed twice in ddH20 and incubated in 60% isopropanol for 5 min at RT and subsequently desiccated in a drying chamber at 45°C for 10 min. Cells were incubated in Oil Red O (ORO) working solution (60% isopropanol) for 10 min at RT and subsequently washed 4× with ddH2O to remove any remaining ORO dye. Once again, cells were completely desiccated in a drying chamber at 45°C for 10 min. An equal volume (1 ml) of 100% isopropanol was added to each well and incubated for 10 min at RT and subsequently extracted for colorimetric analysis. Each sample was analyzed in triplicate using a 96‐well plate reader at 500 nm wavelength.

### 2.8 Fluorescence activated cell sorting analysis

Cells were analyzed for total mitochondrial levels using MitoTracker Red (MTR) CMXRos dye (Thermo Fisher Scientific) and a fluorescence activated cell sorting (FACS) analysis technique. The flow rate was adjusted for a maximum of 2,000 events per second and assessed by a time versus scatter plot to eliminate artifacts caused by poor flow. For each sample 10,000 events were collected, and a FSC versus SSC (FSC/SSC) plot of nontreated, unstained cell culture for initial gated population P1, to exclude debris in sample analysis. Nonstained and single‐stained controls were also used for population gating in sample analysis. Each experiment was performed in biological triplicate on independent days. Temporal analysis of physiological responses of individual cells to stressor exposure was acquired on a BD AccuriTM C6 Plus (BD Biosciences) instrument interfaced with FACS Diva v6.11 software (Becton, Dickinson and Co.) and analyzed using FlowJo v10.2 software (TreeStar Inc.). Instrument acquisition, data analysis, and reporting were carried out as suggested by the International Society for Analytical Cytology (ISAC). Optimal signal‐to‐noise ratio was attained by setting detection threshold voltages in the forward‐scatter (FSC) and side‐scatter (SSC) channels just below that of the lowermost yeast cell signals. For multicolor flow cytometry, nontreated (vehicle), nonstrained, and single‐stained controls were used to correctly adjust hardware and software compensation values. Pretreatment with or without TY addition of stressor were evaluated for cell vitality, mitochondrial membrane potential, cellular oxidative state and ROS production, and features of apoptosis.

### 2.9 Statistical analysis

Individual culture experiments were performed in triplicate and repeated using matched controls, and the data were pooled. Results are expressed as mean ± standard error of the mean (*SEM*). The statistical significance of differences (*p* < .05) was assessed by student's *t* test or a one‐way ANOVA with subsequent post hoc analysis, as appropriate.

## 3 RESULTS

### 3.1 Caffeine increased fatty utilization without negatively impacting mitochondrial oxygen consumption

**Figure 1:**
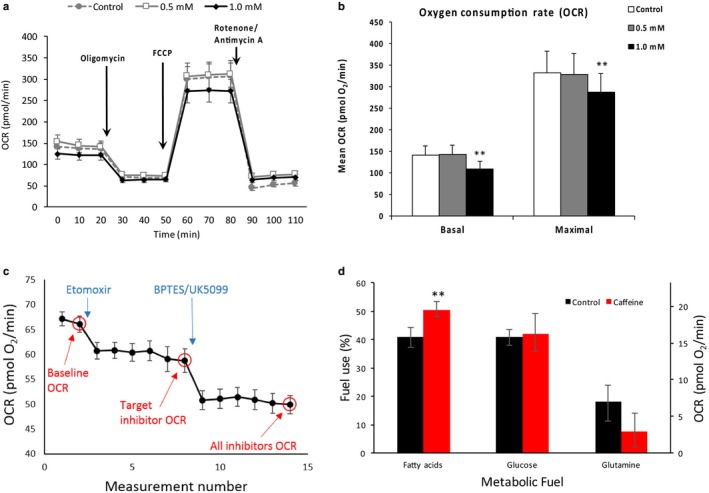
Dose dependence of caffeine reduction of basal and maximal oxygen consumption rate (OCR) and increase in fatty acid metabolism in murine skeletal myotubes. C2C12 skeletal myotubes were treated with vehicle, 0.5, and 1.0 mM caffeine for 24 hr (*n* = 3). Basal and maximal OCR were measured using Seahorse Metabolic Flux Analysis. (a–b) A caffeine dose of 1.0 mM, but not 0.5 mM, promoted a 25% reduction in basal and maximal OCR in murine skeletal myotubes. (c–d). The administration of 0.5 mM caffeine for 24 hr promoted a 25% increase in OCR attributed to fatty acid oxidation in 0.5 mM oleic acid OA‐treated myotubes versus cells treated with 0.5 mM OA alone (*n* = 3). **p < .5

To examine the dose‐dependent effect of caffeine on mitochondrial oxygen consumption, we conducted real‐time analysis of oxygen consumption rate (OCR) in control, 0.5, and 1.0 mM caffeine‐treated cells upon 24 hr incubation. Our analysis revealed that 1.0 mM caffeine reduced basal and maximal OCR by approximately 15% (*p* < .05) versus control cells (Figure 1a and b). However, 0.5 mM caffeine did not significantly affect basal or maximal OCR in skeletal myotubes. To determine whether 0.5 mM caffeine could significantly increase fatty acid metabolism, we conducted additional experiments to measure the mitochondrial OCR attributed to fatty acid, glucose, and glutamine utilization. Caffeine significantly increased the relative proportion of fatty acid utilization by 25% (*p* < .05) when compared to control cells (Figure 1c and d). Our analysis also revealed that total glucose and glutamine utilization were not significantly affected by the addition of caffeine. These results indicate that treating skeletal myotubes with 0.5 mM caffeine for 24 hr significantly increased fatty acid utilization without negatively impacting mitochondrial function.

### 3.2 Caffeine promoted autophagy in a dose‐dependent manner in skeletal myotubes

**Figure 2:**
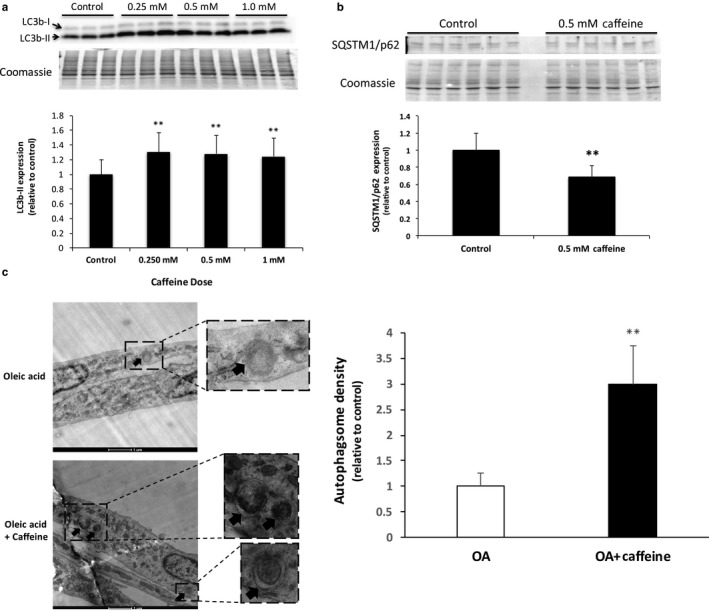
Low‐dose caffeine administration increased autophagy in murine skeletal myotubes. C2C12 skeletal myotubes were incubated with vehicle (control), 0.25, 0.5, or 1.0 mM caffeine for 24 hr. (a) Western blot analysis (*n* = 6) revealed that 0.25–1.0 mM caffeine promoted an approximate 25% increase in the expression of the autophagosome marker microtubule‐associated proteins 1A/1B light chain 3B‐II (LC3b‐II) (*n* = 6). (b) Western blot analysis also revealed a 40% reduction in sequestosome‐1/ubiquitin‐binding protein p62 (SQSTM1/p62) myotubes treated with 0.5 mM caffeine versus those treated with vehicle only. A reduction in SQSTM1/p62 levels is indicative of increased lysosomal degradation of autophagosome cargo, or autophagic flux (*n* = 6). (c) Transmission electron microscopy analysis revealed a twofold increase in autophagosome formation in myotubes‐treated 0.5 mM oleic acid (OA) +0.5 mM caffeine for 24 hr versus cells treated with 0.5 mM OA alone (*n* = 3). **p < .05

To determine the dose‐dependent effect of caffeine on autophagy induction in skeletal muscle cells, we treated C2C12 skeletal myotubes with 0.25, 0.5, or 1.0 mM caffeine for 24 hr. Western blot analysis revealed that treating C2C12 murine skeletal myotubes with all three caffeine doses promoted an approximate 25% increase in the autophagosome marker LC3b‐II (Figure 2a; *p* < .05). To further examine the effect of 0.5 mM caffeine on autophagy activation in skeletal myotubes, we examined the expression of the autophagic flux marker Sequestosome‐1/p62 (SQSTM1/p62). Caffeine promoted a 40% (*p* < .05) reduction in SQSTM1/p62 levels, indicating a significant increase in autophagic flux in caffeine versus control myotubes (Figure 2b). Finally, to confirm our western blot results and to determine whether 0.5 mM caffeine could significantly increase autophagy under high fatty acid conditions, myotubes were incubated in media supplemented with 0.5 mM OA for 24 hr followed by an additional 24 hr incubation in media containing 0.5 mM OA plus vehicle or 0.5 mM caffeine. TEM analysis revealed a twofold increase in autophagosome accumulation in caffeine‐treated versus control cells (Figure 2c; *p* < .05). These results demonstrate that 0.5 mM caffeine promoted a significant increase in autophagy in murine skeletal myotubes.

### 3.3 Caffeine‐dependent lipid utilization was partially dependent on autophagy in skeletal myotubes

**Figure 3:**
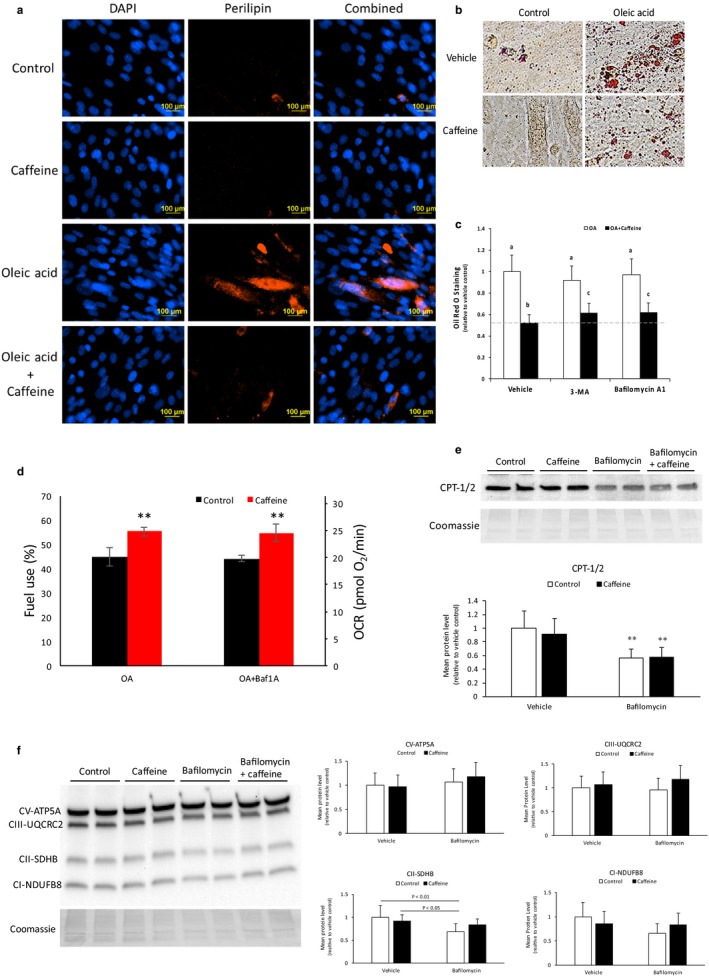
Caffeine promotes autophagy‐dependent lipid utilization in murine skeletal myotubes. C2C12 skeletal myotubes were incubated with vehicle (control) or 0.5 mM oleic acid (OA) for 24 hr. Myotubes were subsequently incubated in either vehicle or 0.5 mM (OA) for an additional 24 hr in the presence or absence of 0.5 mM caffeine. (a) Immunofluorescence microscopy analysis revealed that the addition of OA promoted a 2.5‐fold increase in perilipin levels, which reduced to control levels by the addition caffeine (*n* = 3), (b) which was confirmed by Oil Red O (ORO) analysis. (c) Further ORO analysis revealed that the addition of autophagy inhibitors 3‐MA (10 mM) or Bafilomycin A1 (4 μM) reduced caffeine‐dependent lipid utilization by approximately 30% (*n* = 6). (d) Seahorse Metabolic Flux Analysis reveal that 0.5 mM caffeine promoted an approximate 25% increase fatty acid utilization (*n* = 3). (e) Western blot analysis revealed a 45% reduction in carnitine palmitoyltransferase‐1/2 (CPT‐1/2) levels in bafilomycin‐treated cells. (f) Western blot analysis of mitochondrial Complex I subunit NDUFB8 (CI‐NDUFB8), Complex II SDHB (CII‐SDHB), Complex III‐Core protein 2 (CIII‐UQCRC2), and Complex V alpha subunit (CV‐ATP5A) revealed a significant reduction in the level of CII‐SDHB in cells treated with bafilomycin alone. ^a,b,c^Indicate significant difference between treatments without same letters (**p < .05)

To further examine the effect of caffeine on skeletal muscle lipid metabolism, we incubated myotubes in 0.5 mM OA for 24 hr followed by an additional 24 hr incubation in media containing 0.5 mM OA in the presence or absence of 0.5 mM caffeine. Immunocytochemistry analysis revealed a 2.5‐fold increase in perilipin staining in skeletal myotubes treated with 0.5 mM OA versus nontreated cells (Figure 3a; *p* < .05). The addition of caffeine reduced the level of Perilipin‐1 staining to control levels (Figure 3a; *p* < .05), indicating the strong lipolytic effect of 0.5 mM caffeine in skeletal myotubes. These results were subsequently confirmed using ORO analysis (Figure 3b; *p* < .05). To determine whether caffeine‐increased lipid utilization in an autophagy‐dependent manner, we treated skeletal myotubes with specific autophagy inhibitors 3‐methlyadenine (3‐MA; 10 μM) or bafilomycin A1 (4 μM). Oil Red O analysis revealed that the addition of either of 3‐MA or bafilomycin A1 reduced the caffeine‐dependent decrease in lipid accumulation by approximately 30% (Figure 3c; *p* < .05). These results demonstrate that the caffeine‐dependent decrease in lipid accumulation in the presence of OA was partially dependent upon autophagy in skeletal myotubes. However, OCR measurements indicated that the caffeine‐dependent increase in relative fatty acid oxidation was not altered by the addition of bafilomycin (Figure 3d). To further examine the effect of autophagy inhibition on mitochondrial fatty acid metabolism, we conducted western blot analysis of CPT‐1/2 and total OXPHOS proteins (Figure 3e‐f). Caffeine alone did not significantly impact CPT‐1/2 levels, however, the addition bafilomycin reduced CPT‐1/2 by approximately 45% in control and caffeine‐treated cells (Figure 3e; *p* < .05). Total OXPHOS proteins were analyzed using a commercially available cocktail that contained primary antibodies for Complex I subunit NDUFB8 (CI‐NDUFB8), Complex II SDHB (CII‐SDHB), Complex III‐Core protein 2 (CIII‐UQCRC2), and Complex V alpha subunit (CV‐ATP5A). Once again, caffeine alone did not significantly impact the expression any OXPHOS proteins. However, the addition of bafilomycin significantly reduced the level of CII‐SDHB in cells treated with bafilomycin alone (Figure 3f; *p* < .05). Although autophagy appears to account for some of the caffeine‐dependent decrease in cellular lipid content, it did not directly impact total fatty acid oxidation in skeletal myotubes.

### 3.4 Caffeine did not increase lipophagy in skeletal myotubes

**Figure 4:**
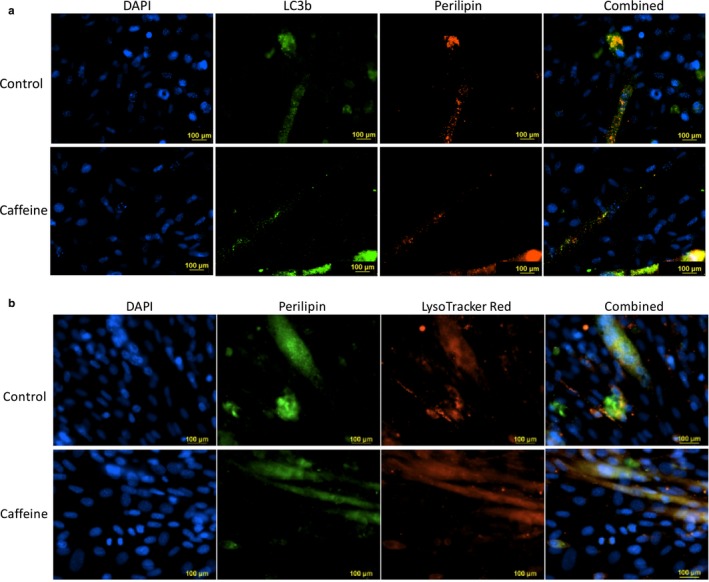
Caffeine did not significantly increase the association of lipid droplets with autophagosomes or lysosomes in murine skeletal myotubes. C2C12 skeletal myotubes were treated with 0.5 mM OA for 24 hr, followed by an additional 24 hr incubation in 0.5 mM OA with the addition of vehicle or 0.5 mM caffeine. Cells were colabeled with primary antibodies for (a) LC3b and perilipin or (b) LysoTracker Red and perilipin. Colocalization analysis revealed that caffeine did not significantly increase the Pearson's coefficient between LC3b and perilipin or LysoTracker Red and perilipin (*p* > .05)

To determine whether caffeine promoted lipophagy in skeletal myotubes, we conducted fluorescence microscopy and colocalization analysis. Once again, myotubes were treated with 0.5 mM OA for 24 hr, followed by an additional 24 hr incubation in 0.5 mM OA with the addition of vehicle or 0.5 mM caffeine. Cells were colabeled with primary antibodies for LC3b and Perilipin‐1 or LysoTracker Red and Perilipin‐1 (Figure 4). Colocalization analysis revealed that caffeine did not significantly increase the Pearson's coefficient between LC3b and Perilipin‐1 or LysoTracker Red and Perilipin‐1 (Figure 4). These results strongly suggest that caffeine did not significantly increase the association of lipid droplets with autophagosomes or lysosomes. To further examine the effect of caffeine on lipophagy in skeletal myotubes, we conducted TEM analysis using the same experimental design. We did not find a significant increase in lipid droplets contained in autophagosomes or autolysosomes in caffeine versus vehicle‐treated cells (data not shown). Our results strongly suggest that while caffeine increased autophagy, it did not increase lipophagy per se in skeletal myotubes.

### 3.5 Caffeine significantly increased mitochondrial turnover and fragmentation in skeletal myotubes

**Figure 5:**
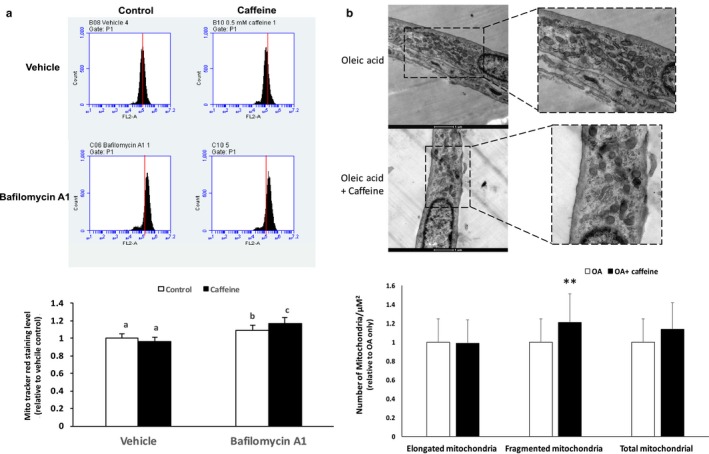
Caffeine increases mitochondrial turnover with impacting mitochondrial density in murine skeletal myotubes. C2C12 skeletal myotubes were treated with 0.5 mM OA for 24 hr. Control and 0.5 mM caffeine‐treated cells received either (DMSO) or 4 μM bafilomycin A1 for an additional 24 hr. Following Mito Tracker Red (MTR) labeling, total fluorescence was measured using FACS analysis. (a) Caffeine alone did not significantly alter mitochondrial density as there was not a significant difference in MTR fluorescence. The addition of bafilomycin A1 promoted a 10% and 18% increase in MTR fluorescence in vehicle and caffeine‐treated cells, respectively. (b) Transmission electron microscopy (TEM) analysis was conducted on skeletal myotubes treated with 0.5 mM OA or 0.5 mM OA plus 0.5 mM caffeine for 24 hr. TEM analysis revealed a 35% increase in mitochondrial fragmentation. ^a,b,c^Indicate significant difference between treatments without same letters (**p < .05)

To examine the effect of caffeine on mitochondrial turnover, we added vehicle (DMSO) or 4 μM bafilomycin A1 to control and 0.5 mM caffeine‐treated cells for 24 hr. Following MTR labeling, total fluorescence was measured using FACS analysis. Caffeine alone did not significantly alter mitochondrial density as there was not a significant difference in MTR fluorescence in control versus caffeine‐treated cells (Figure 5a). However, the addition of bafilomycin A1 promoted a 10% (*p* < .05) and 18% (*p* < .05) increase in MTR fluorescence in vehicle and caffeine‐treated cells, respectively (Figure 5a). These results together suggest that caffeine increased the rate of mitochondrial degradation, but this was offset by an increase in mitochondrial biogenesis, leading to no net change in mitochondrial density. These results are consistent with the absence of an effect of 0.5 mM caffeine on basal or maximal OCR (Figure 1a and b). To further assess the effect of caffeine on mitochondrial biogenesis, we treated skeletal myotubes with 0.5 mM OA or 0.5 mM OA plus 0.5 mM caffeine for 24 hr. TEM analysis revealed that caffeine did not significantly increase the number of elongated mitochondria or total mitochondrial density (Figure 5b). However, caffeine did promote a 35% increase in mitochondrial fragmentation (Figure 5b). These results strongly suggest that caffeine significantly increased mitochondrial turnover in skeletal myotubes without significantly impacting total mitochondrial density.

## 4 DISCUSSION

The popular dietary stimulant caffeine has been shown to increase lipid utilization, mitochondrial biogenesis, and autophagy in skeletal muscle cells treated with very high doses of caffeine (Abbott, Edelman, & Turcotte, 2009; Ding et al., 2012; Hughes et al., 2017; Mathew et al., 2014). It remains unclear whether lower, more physiologically relevant doses of caffeine can similarly impact lipid metabolism and autophagy in mammalian skeletal muscle cells. The purpose of this study was to determine whether a more physiologically relevant dose of caffeine could increase lipid utilization in an autophagy‐dependent manner. Furthermore, we sought to determine if caffeine could increase mitochondrial turnover in skeletal muscle cells. Our first objective was to examine the dose‐dependent effect of caffeine on mitochondrial metabolism. We discovered that 1.0 mM caffeine negatively impacted basal and maximal OCR (Figure 1). However, 0.5 mM caffeine had no impact on mitochondrial OCR. Our results directly contrast those of Vaughan, Garcia‐Smith, Bisoffi, Trujillo, and Conn (2012), who discovered a twofold increase in basal and fivefold increase in maximal OCR in human rhabdomyosarcoma cells treated with 0.5 mM caffeine for 24 hr. Vaughan et al. (2012) attributed the caffeine‐dependent increase in basal and maximal OCR to a 25% increase in mitochondrial content (density) in caffeine versus control cells. As previously stated, we found that caffeine did not significantly increase mitochondrial density in skeletal myotubes (Figure 5), which most likely accounts for the lack of a caffeine‐dependent increase in mitochondrial OCR in this study. We further examined the effect of 0.5 mM caffeine on aerobic fuel utilization in skeletal myotubes treated with 0.5 mM OA. Caffeine promoted a 25% increase in relative fatty acid oxidation without impacting glucose or glutamine utilization (Figure 1). These results are similar to those of previous investigations that reported the lipolytic effects of caffeine in skeletal muscle cells (Abbott et al., 2009). To the best of our knowledge, however, this investigation is first to report that a physiologically relevant dose of caffeine can increase fatty acid utilization in skeletal myotubes cells without impacting basal or maximal rates of aerobic metabolism. This is consistent with the notion that metabolic rate responds to changes in ATP demand. Thus, a shift toward increased fatty acid oxidation would not be expected to alter OCR unless ATP demand was also changed. This view is supported by Figure 1d, which shows that the increase in fatty acid oxidation is offset by a comparable (although nonsignificant) decrease in glutamine oxidation.

Our next objective was to determine whether 0.5 mM caffeine could increase autophagy in control and OA‐treated skeletal muscle cells. We discovered that treating control myotubes with 0.25–1.0 mM caffeine promoted a 25% increase in autophagosome formation and that 0.5 mM caffeine promoted a 40% increase in autophagic flux as indicated by reduced SQSTM1/p62 levels (Figure 2). Furthermore, we discovered that 0.5 mM caffeine promoted a twofold increase in autophagosome accumulation in 0.5 mM OA‐treated cells (Figure 2). Although we have previously demonstrated that caffeine increases autophagy in skeletal myotubes (Hughes et al., 2017; Mathew et al., 2014), to the best of our knowledge this is the first investigation to demonstrate that a physiologically relevant dose of caffeine can significantly increase autophagy in mammalian skeletal myotubes.

Next, we sought to determine whether caffeine increased lipid utilization in an autophagy‐dependent manner. To determine the magnitude of lipid uptake and utilization in skeletal myotubes, we conducted fluorescent microscopy and ORO analysis. Fluorescent microscopy analysis revealed that treating skeletal myotubes with 0.5 mM OA for 24 hr promoted a 2.5‐fold increase in Perilipin‐1 staining, which was reduced to control levels by the addition for 0.5 mM caffeine for an additional 24 hr (Figure 3). These results were verified using ORO analysis. To determine whether caffeine promoted lipid utilization in an autophagy‐dependent manner, we treated skeletal myotubes with the early‐stage autophagy inhibitor 3‐MA or the late‐stage autophagy inhibitor bafilomycin A1. The addition of either autophagy inhibitor promoted a 30% reduction in caffeine‐dependent decrease in lipid accumulation, as determined by ORO analysis (Figure 3c). However, colocalization analysis did not reveal a significant difference in the correlation between Perilipin‐1 and LC3b or Perilipin‐1 and Lyso Tracker Red labeled lysosomes in caffeine versus control cells (Figure 4). Furthermore, TEM analysis did not reveal a significant increase in the presence of lipid droplets sequestered in autophagosomes or autolysosomes in caffeine versus control cells (data not shown). In addition, bafilomycin A1 did not lead to a reduction in fatty acid oxidation (Figure 3d). Interestingly, the addition of bafilomycin significantly reduced CPT‐1/2 level in control and caffeine‐treated cells (Figure 3e). These results are consistent with previous investigations that demonstrated the necessity of autophagy for peroxisome proliferator‐activated receptor alpha (PPAR alpha)‐regulated expression of CPT‐1 (Saito et al., 2019; Song et al., 2010). Given the importance of CPT‐1 in regulating mitochondrial fatty acid metabolism, it is interesting that autophagy inhibition did not impact total fatty acid oxidation levels. Further research is needed to determine the mechanism by which caffeine increases mitochondrial fatty acid oxidation in skeletal muscle cells.

Our results clearly indicate that the caffeine‐dependent decrease in lipid stores in skeletal myotubes is partially dependent upon autophagy activation, however, we did not find direct evidence of increased lipophagy in caffeine‐treated cells. Most previous investigations that have linked autophagy with increased lipid metabolism in mammalian cells have reported direct evidence of lipophagy (Ro et al., 2013; Singh et al., 2009; Zhang et al., 2018). Because we found no direct evidence of caffeine‐dependent lipophagy, we were forced to explore other autophagy‐dependent pathways that could potentially contribute to increased lipid accumulation.

Accelerated oxidative metabolism has also been linked to increased mitochondrial turnover. Melser et al. (2013) recently demonstrated that providing increased glutamine concentrations in the absence of glucose significantly increased mitochondrial metabolism in human primary skeletal muscle myoblasts. This increase in mitochondrial metabolism was dependent upon increased mitochondrial turnover as autophagy inhibition, and thus mitochondrial turnover, completely prevented the increase in mitochondrial metabolism (Melser et al., 2013). Similarly, Singh et al. (2018) discovered that the ability of thyroid hormone (T3) to increase mitochondrial metabolism was entirely dependent upon increased mitochondrial turnover. In this study, flow cytometry revealed an 80% increase in basal mitochondria levels in caffeine versus control cells in the presence, but not in the absence, of bafilomycin A1 (Figure 4). These results strongly suggest that caffeine increased mitochondrial turnover in skeletal myotubes. As previously indicated, an identical dose of bafilomycin A1 reduced caffeine‐dependent lipid utilization by approx. 30% (Figure 3). Therefore, we propose that the autophagy‐dependent component of caffeine‐induced lipid utilization in skeletal myotubes was most likely due to increased mitochondrial turnover rather than a direct increase in lipophagy. In addition, we discovered that even though caffeine did not impact total mitochondrial density, it did significantly increase the number of fragmented mitochondria (Figure 5). Increased mitochondrial fragmentation has been shown to precede and to be necessary for increased mitophagy under certain conditions (Frank et al., 2012; Twig et al., 2008). Therefore, our results suggest that caffeine increased mitochondrial turnover in skeletal myotubes, which likely accounted for the autophagy‐dependent component of the caffeine‐induced decrease in lipid content.

The caffeine concentration used in this manuscript reflects a physiologically relevant dose. A caffeine concentration of 0.5 mM is equivalent to approximately 100 mg/kg of body mass. Using the model proposed by Reagan‐Shaw, Nihal, and Ahmad (2008), a 100 mg/kg dose of caffeine in murine skeletal muscle is equivalent to approximately 8 mg/kg (40 µM) in human skeletal muscle. Highly caffeinated beverages, including coffee and tea, contain approximately 50–100 mg per 8‐ounce serving (1–2 mM).

Therefore, our results are possibly indicative of the metabolic benefits of consuming multiple servings of highly caffeinated beverages, such as coffee or tea. Green tea has been shown to be especially beneficial in reducing the risk of metabolic disorders, including abdominal obesity (Bhatti, O'Keefe, & Lavie, 2013). In present study, we demonstrate that a physiologically relevant dose of caffeine can increase fatty acid oxidation and reduce lipid accumulation in skeletal muscle cells. Future studies are needed to determine the role of autophagy in regulating the caffeine‐dependent increase in lipid metabolism in skeletal muscle cells.

## 5 CONCLUSION

In conclusion, our results strongly suggest that caffeine increased lipid utilization in skeletal myotubes in a manner that was partially dependent on autophagy activation. Furthermore, our results suggest that caffeine did not significantly increase lipophagy, but rather it promoted a significant increase in mitochondrial turnover. Further research is needed to conclusively determine whether a physiologically relevant dose of caffeine can increase mitochondrial turnover in skeletal muscle cells in vivo.

## CONFLICT OF INTEREST

None.
